# Boosting cancer therapy with self-assembled inorganic nanocarriers via host-guest chemistry

**DOI:** 10.1016/j.mtbio.2026.102901

**Published:** 2026-02-07

**Authors:** Ying Tang, Yifei Mao, Luxi Wang, Yuan-Fu Ding, Peng Li, Beibei Xie

**Affiliations:** a State Key Laboratory of Mechanism and Quality of Chinese Medicine, Institute of Chinese Medical Sciences, University of Macau, 999078, Macao; b School of Medical and Information Engineering, Scientific Research Center, Gannan Medical University, Ganzhou, 341000, China

**Keywords:** Inorganic nanocarriers, Host-guest interaction, Supramolecular self-assembly, Cancer therapy

## Abstract

Inorganic nanocarriers (INCs) have been widely used in drug delivery systems due to their excellent biocompatibility, ease of synthesis and functionalization, cost-effectiveness, and robust physicochemical stability. However, small-sized INCs are prone to rapid in vivo clearance, resulting in low accumulation and limited retention in target tissues or cells, significantly reducing their therapeutic efficacy. The self-assembly of small INCs into large aggregates represents an innovative approach for enhancing their tumor-specific accumulation and retention. This review primarily focuses on the stimuli-responsive self-assembly of INCs, including gold nanoparticles, calcium carbonate nanoparticles, ferroferric oxide nanoparticles, and silica nanoparticles, driven by supramolecular host-guest interactions. Specifically, this strategy aims to increase accumulation concentrations, prolong retention time, and achieve precise spatiotemporal control over drug release for tumor treatment. Thus, this review provides a critical summary and reference for exploring supramolecular host-guest interactions in drug delivery systems and promotes the advancement of supramolecular chemistry as an emerging interdisciplinary field.

## **Graphical abstract**

This review summarizes the most recent progress on the self-assembly of versatile inorganic nanocarriers via host-guest interactions in cancer therapeutics.

**Figure:**
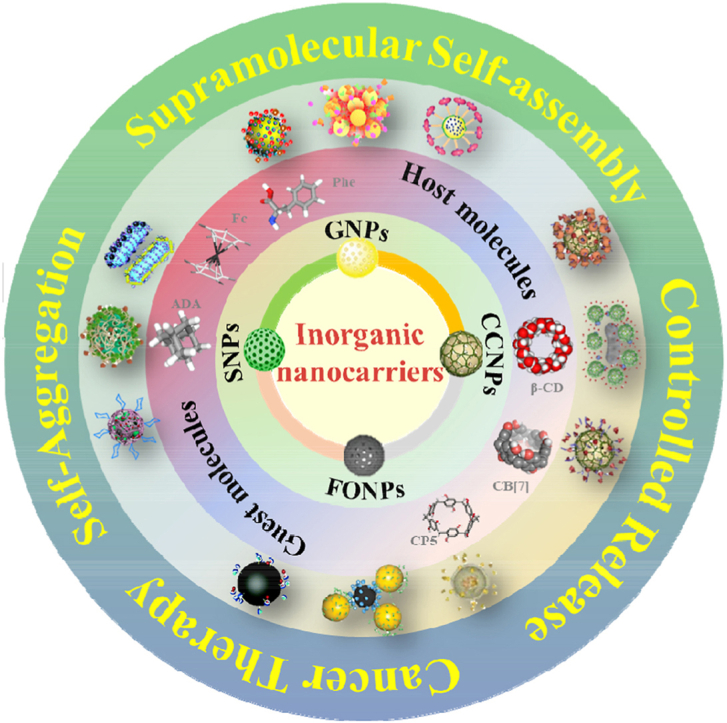

**Table:** List of abbreviations

ADA	Adamantane
ADA-COOH	ADA carboxylic acid
APD	Aminopyridine
APTES	3-Aminopropyltriethoxysilane
Azo	Azobenzene
BM-MHMSNs	Benzimidazole -modified magnetic hollow MSNs
Bzl	Benzimidazole
c(RGDfC)	Cyclo(Arg-Gly-Asp-d-Phe-Cys)
CB[6]	Cucurbit[6]uril
CB[7]	Cucurbit[7]uril
CB[8]	Cucurbit[8]uril
CCNPs	Calcium carbonate nanoparticles
CD	Cyclodextrin
CD-SH	Cyclodextrin-thiol
CM	Cell membrane
CP5	Carboxylate pillar[5]arene
DC	Dendritic cell
DOX	Doxorubicin
DSPE-PEG-CB	1,2-Distearoyl-sn-glycero-3-phosphoethanolamine-poly(ethyleneglycol)-CB
EDA	Ethylenediamine
EPR	Enhanced permeability and retention
Fc	Ferrocene
FFVLK	Phe-Phe-Val-Leu-Lys
F-Kyn	N-formyl kynurenine
FONPs	Ferroferric oxide nanoparticles
FPC	Folate-conjugated polyethyleneimine-β-CD
FRs	Folate receptors
GMA	poly(glycidyl methacrylate)
GNPs	Gold nanoparticles
GNRs	Gold nanorods
GPX4	Glutathione peroxidase 4
GSH	Glutathione
H_2_O_2_	Hydrogen peroxide
HA	Hyaluronate
HA	Hyaluronic acid
HCC	Hepatocellular carcinoma cells
IDO1	Indoleamine 2,3-dioxygenase 1
INCs	Inorganic nanocarriers
LSPR	localized surface plasmon resonance
MRI	Magnetic resonance imaging
MSNs	Mesoporous silica nanoparticles
MV^2+^	Methyl viologen
NADPH	Nicotinamide Adenine Dinucleotide Phosphate
NIR	Near-infrared
O_2_•^-^	Superoxide anion radical
OMVs	Outer membrane vesicles
OX	Oxaliplatin
PEG	Polyethylene glycol
PGED-CD	CD-conjugated PGED
Phe	Phenylalanine
PNIPAm	poly(N-isopropylacrylamide)
PTT	Photothermal therapy
Py	Pyridinium
RBC	Red blood cells
ROS	Reactive oxygen species
SAS	Sulfasalazine
SNPs	Silica nanoparticles
SPM	Spermine
system Xc^-^	Cystine/glutamate antiporter
TME	Tumor microenvironment
Trp	Tryptophan
UV	Ultraviolet
VNP	Salmonella typhimurium VNP20009
α/β-HPCD	α/β Hydroxypropyl cyclodextrin
β-CD	β-cyclodextrin
•OH	Hydroxyl radical
^1^O_2_	Singlet oxygen
2-NAA	2-Naphthaleneacetic acid

## 1 Introduction

Cancer is one of the leading causes of death globally, with approximately 18.74 million new cases diagnosed and about 9.74 million fatalities annually [1,2]. The increased incidence and mortality rates of cancer pose significant global challenges to achieve long life expectancy, bring an urgent demand for ground-breaking innovations and ever-improving tactics. Chemotherapy based on chemical drugs remains a major tool for the therapy of cancer in clinics [3]. However, the lack of specificity of chemotherapeutic drugs towards tumor cells often results in severe side effects and suboptimal efficacy.

The advent of nano-encapsulation-based drug delivery systems could significantly improve the delivery efficiency and stability of chemotherapeutic drugs [4,5]. Notably, inorganic nanocarriers (INCs) have been widely utilized in drug delivery systems due to their ease of synthesis, low cost, stable physical and chemical properties, and exceptional resistance to hydrolytic or enzymatic degradation [6,7]. INCs possess unique physicochemical properties, including a high surface area per unit volume, distinctive optical and magnetic properties. Moreover, INCs could be functionalized with a variety of specific ligands to enhance their affinity for target cells or molecules. Beyond enabling controlled drug release, INCs also could protect drugs from degradation, which not only reduces the frequency of administration and the required drug dosage but also leads to a significant decrease in drug toxicity [8].

Representative INCs include gold nanoparticles (GNPs) [9], calcium carbonate nanoparticles (CCNPs) [10], ferroferric oxide nanoparticles (FONPs) [11], and silica nanoparticles (SNPs) [12]. GNPs exhibit the capability to convert light into both acoustic signals and heat, thereby enabling their application in imaging-guided photothermal therapy (PTT) [[13], [14], [15]]. CCNPs decompose slowly at normal physiological pH but fast under acidic tumor microenvironment (TME) [16,17]. FONPs with magnetism could be magnetically targeted to tumor sites and assist in magnetic resonance imaging (MRI) to trigger ferroptosis [18,19]. SNPs with pore sizes ranging from 2 nm to 50 nm are excellent candidates for drug delivery and biomedical applications [20,21]. Despite these advantages, INCs with a small size are prone to rapid clearance, leading to inadequate accumulation and short retention times in targeted tissues and cells, weakening the treatment effects [22,23].

To address these challenges, scientists utilize the TME to induce the self-assembly of small-sized INCs into larger aggregates at the tumor site, which can effectively reduce clearance, enhance accumulation, and prolong retention of INCs. The self-assembly of INCs highly depends on ligands modified on their surfaces and induced by exogenous stimuli such as light [24] and temperature [25], or endogenous stimuli such as acidic conditions [26] and enzymes [27], to prompt their aggregation at the tumor site [28]. The key driving forces for the aggregation of INCs are mainly covalent condensation reactions and non-covalent interactions [29]. However, self-assembly methods based on covalent bonding or electrostatic interactions are irreversible, complex and uncontrollable, which may lead to delayed clearance from the body.

Supramolecular chemistry, a field established by Nobel laureate Jean-Marie Lehn in 1987 [30], mainly focuses on the organized assembly of molecular subunits through reversible non-covalent interactions, resulting in complex structures with novel or enhanced functions [31]. In 1967, Pedersen first reported artificial host-guest complexes using crown ethers, a class of macrocyclic hosts to recognize alkali metal cations as guests selectively [32]. Unlike traditional covalent chemistry, host-guest chemistry relies on dynamic non-covalent interactions, including hydrophobic effects, hydrogen bonding, π-π interactions, and van der Waals forces [33] and exhibits. The combination of multiple interaction sites and non-covalent bonds can collectively stabilize host-guest complexes [34]. Compared with self-assembly based on covalent bonding, the self-assembly methods based on host-guest interaction possess some advantages, including dynamic reversibility, excellent biocompatibility, stimulus responsiveness, and high design flexibility [35]. This positive synergistic effect endows supramolecular self-assembly with unlimited potentialin overcoming the limitations of covalently-based self-assembly.

The development of various macrocyclic host molecules, such as cyclodextrins (CDs), cucurbit[n]urils (CB[n]s), calix[n]arenes, pillar[n]arenes, crown ethers, cyclophanes, and cryptands [36], promotes the wide application of supramolecular chemistry in the field of biomedicine. The rigid hydrophobic cavities with distinct sizes and molecular recognition properties of hosts enable precise encapsulation and release of drugs within tumors, achieving efficient therapy of tumors with mild side effects. Combining self-assembly strategies based on supramolecular chemistry with INCs can promote tumor-specific drug accumulation and enable stimuli-responsive drug release, ultimately improving therapeutic outcomes [37,38].

By integrating supramolecular strategies with INCs , researchers are developing safer and more effective cancer treatments to overcome the limitations of conventional chemotherapy. Therefore, utilizing host-guest interactions to guide the self-assembly of INCs can promote tumor-specific drug accumulation and enable stimuli-responsive drug release, ultimately improving therapeutic outcomes.

**Fig. 1:**
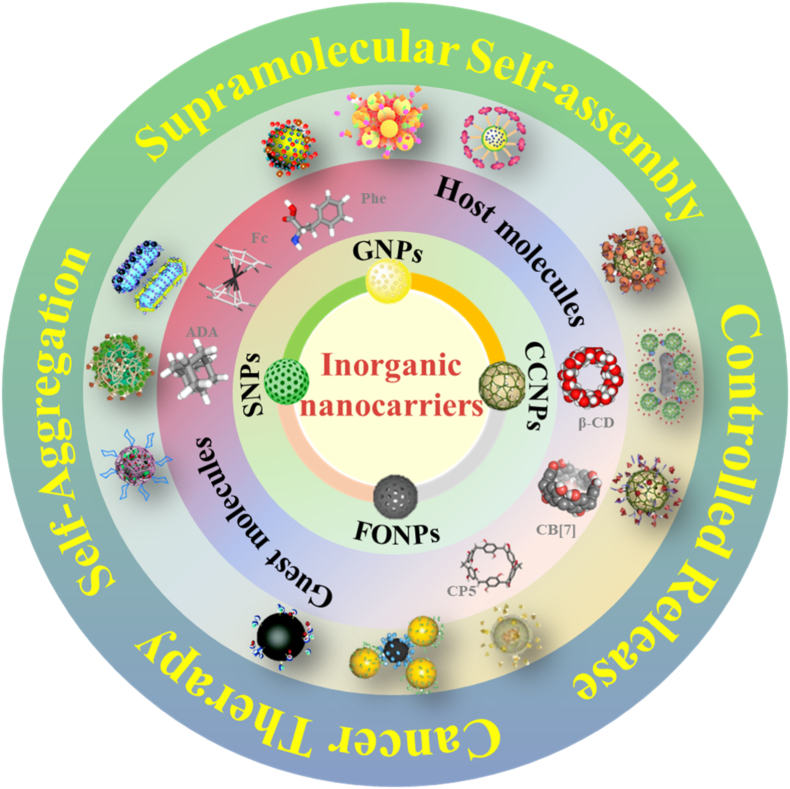
Schematic illustration of self-assembly of INCs mediated by supramolecular host-guest interactions.

In this review, we summarize the most recent progress on the self-assembly of versatile INCs, including GNPs, CCNPs, FONPs, and SNPs via host-guest interactions in cancer therapeutics (Fig. 1). By explaining the design rules and biological effects unique to each type, this review creates a blueprint for smart drug systemsto control drugs release and improve treatment efficiency (Table 1).

**Table 1:** Representative INCs leveraging host-guest interactions in recent years.

INCs	Host molecules	Guest molecules	Accumulation at tumor site/Drug release mechanisms	Therapeutic outcomes	Ref.
GNPs	CD-SH	ADA-PEG-SH	Noninvasive PTT	High tumor-targeting efficiency (>10.5% ID/g); Enhanced NIR absorption	[39]
	β-CD-SH	2-NAA-HA conjugate	Light-triggered drug release; synergistic photothermal-chemotherapy	Excellent stability, high biocompatibility, improved tumor-cell selectivity	[40]
	β-CD-SH	ADA-SH	PTT/immunotherapy	Minimal premature drug loss; enhanced targeting efficiency	[41]
	CB[7]	Quaternary ammonium-thiol	Regulate the exocytosis of GNPs	Prolonged intracellular retention of drug carriers	[42]
	β-CD-SH	ADA-PEG; ADA-PNIPAm	Drug release with a decrease in temperature	Localized drug release	[43]
CCNPs	Engineered living bacteria-DSPE-PEG-CB	ADA-COOH	Ca^2+^ overload & immunotherapy; pH-responsive release	Excellent tumor-targeting and colonization ability; potent anticancer activity with minimal side effects	[44]
	β-CD	Tocopherol acetate	pH- or redox-stimulated release	Capable of co-loading three different probes	[45]
	HA-CB[6]	Hydrophilic/hydrophobic model drugs	pH- and ROS-responsive release	Biodegradable nanomotor; targeted drug delivery	[46]
	α/β-HPCD	Pd(II) complex	UV-light-triggered release	Novel catalytic system	[47]
	CB[7]	FFVLK peptide	SPM-responsive release	Induces intracellular biomineralization and Ca^2+^ overload in tumor cells	[48]
FONPs	β-CD	APD	pH-sensitive drug carrier	High stability, good magnetic responsiveness, cytocompatibility	[49]
	CD-COOH	ADA-COOH	Cancer-cell-membrane-targeted supramolecular aggregation	Effective cellular uptake and precise accumulation in tumor cells/tissues	[50]
	β-CD-GMA	ADA	PTT/gene therapy	Bowl-shaped magnetic assembly; efficient gene carrier and MRI contrast agent	[51]
	α-CD	Poly(N-phenylglycine)-PEG	ROS- and NIR-responsive release	In situ-formable supramolecular hydrogel	[52]
	β-CD-SH	Diclofenac	Host-guest-mediated loading and release of hydrophobic drugs	Polydopamine coating; initial burst release within 8 h	[53]
SNPs	β-CD	Azo	Light-responsive release	Rapid and facile preparation	[54]
	β-CD	Fc	Redox-responsive release	Hollow mesoporous silica structure	[55]
	α-CD	PEG	Novel hydrogel-based delivery system	Integration of organic and inorganic components	[56]
	CP5	Py	Multi-stimuli responsive (pH, temperature, EDA, NIR)	Active targeting; quadruple-stimuli-responsive nanogate	[57]
	CD-heparin-PEG	ADA	Redox-responsive release	Biocompatible nanocarrier; reduced DOX toxicity	[58]

**Abbreviations:** CD-SH, Cyclodextrin-thiol; ADA-PEG-SH, Adamantane-polyethylene glycol-thiol; NIR, Near-infrared; 2-NAA-HA, 2-Naphthaleneacetic acid-hyaluronic acid; CB[7], Cucurbit[7]uril; PNIPAm, poly(*N*-isopropylacrylamide); DSPE-PEG, Distearate phosphatidylethanolamine-polyethylene glycol; HA-CB[6], Hyaluronate-CB[6]; ROS, Reactive oxygen species; α/β-HPCD, α/β-Hydroxypropyl cyclodextrin; FFVLK, Phe-Phe-Val-Leu-Lys; APD, Aminopyridine; GMA, poly(glycidyl methacrylate); Azo, Azobenzene; Fc, Ferrocene; CP5, Carboxylate pillar[5]arene; Py, Pyridinium; EDA, Ethylenediamine; DOX, Doxorubicin.

## 2 Stimulus-responsive self-assembly of INCs

### 2.1 Self-assembly of INCs induced by exogenous stimulus

Before exploring self-assembled INCs, it is essential to understand stimulus-responsive systems under various conditions, as this knowledge supports the rational design of future delivery vehicles. Exogenous stimulus-responsive systems enable INCs to react precisely to external signals, such as light, heat, magnetism, or ultrasound, through their intrinsic properties, allowing real-time drug release control and high therapeutic effects [59]. In photothermal systems, GNPs exhibit a localized surface plasmon resonance (LSPR) effect under near-infrared light, enhancing light absorption and generating heat. The resulting hyperthermia disrupts cell membrane integrity and denatures proteins, leading to irreversible cellular damage [60]. Magnetic-responsive systems rely on FONPscould accumulate in tumor tissue under an external magnetic field, inducing cell death via lipid peroxidation or thermal effects [61]. Photodynamic systems employ photosensitizers embedded in mesoporous SNPs to produce ROS under light irradiation, thereby killing tumor cells [62]. Ultrasound-responsive systems could improve nanoparticle penetration and drug release by means of cavitation effects [63].

### 2.2 Self-assembly of INCs induced by endogenous stimulus

Endogenous stimulation primarily originates from the abnormal physicochemical conditions within tumor cells and TME that differ from those in normal tissues [64]. Specifically, they include low pH, high enzyme activity, overexpressed glutathione (GSH), increased levels of ROS, and overexpreed folate receptors (FRs) and spermine (SPM) (Table 2).

**Table 2:** The differences in biological parameters between normal tissues and pathological tissues.

Biological parameters	Normal tissues	Pathological tissues	Ref.
pH	∼7.4	∼6.5 (tumor extracellular sites, inflammatory tissues);	[65]
		5-6 (endosomes);	
		4-5 (lysosomes);	
		∼6.4 (Golgi apparatus)	
GSH	Low expression	Intracellular: 2-20 mM;	[66]
		Extracellular: 0.5-10 μM	
Specific enzyme	Low expression in intracellular compartments	Overexpressed in intracellular compartments	[67]
ROS	Naturally produced at low levels;	Elevated levels of H_2_O_2_, ^1^O_2_, •OH, and O_2_•^-^ via mitochondrial respiratory chain and NADPH oxidase pathways.	[68]
	Involved in physiological signaling		
FR	Kidney: ∼14.40 pmol/mg protein	Serous tumors: ∼34.31 pmol/mg protein	[69]
	Lung: ∼7.79 pmol/mg protein	Primary endometrioid ovarian tumors: ∼15.66 pmol/mg protein	
	Other tissues: <2 pmol/mg protein	Metastatic ovarian carcinomas: ∼46.36 pmol/mg protein	
SPM	8.82 ± 3.12 nmol/10^10^ RBC	Gastric cancer: 21.59 ± 21.00 nmol/10^10^ RBC	[70]
		Colon cancer: 41.59 ± 37.57 nmol/10^10^ RBC	
		Hepatoma: 20.29 ± 11.52 nmol/10^10^ RBC	
		Pancreatic cancer: 17.48 ± 9.44 nmol/10^10^ RBC	
		Pulmonary cancer: 13.48 ± 5.70 nmol/10^10^ RBC	

pH-responsive systems typically incorporate nanomedicines with tertiary amine groups that acquire positive charges in acidic environments, conferring sensitivity to pH variations. Materials responsive to pH often employ acid-cleavable linkers such as maleimide and cis-aconityl hydrazine [71]. Redox-responsive systems utilize disulfide bonds that are cleaved by elevated GSH levels within the TME, enabling controlled drug release with high stability under physiological conditions. This stability makes them especially suitable for oral formulations, as they withstand the gastrointestinal environment [72]. Enzyme-responsive designs incorporate specific substrates such as protease-cleavable peptides as surface coatings or capping agents, ensuring activation only in the presence of TME-specific enzymes [73]. ROS-responsive systems employ H_2_O_2_-reactive materials such as nitrogen-containing compounds, which release drugs by catalyzing the conversion of H_2_O_2_ into oxygen to disrupt the nanoparticles [68]. Folate-responsive strategies leverage the overexpression of FRs on cancer cells-by using supramolecular materials that bind folate molecules to achieve targeted drug delivery [74]. The SPM-responsive system achieves controlled release of drugs based on the difference in affinity between host and guest molecules. In summary, the above-mentioned TME-responsive strategies significantly improve the therapeutic efficiency of drug delivery by specifically adapting to the biological characteristics of the cancer.

As shown in representative INCs constructed through host-guest interactions in recent years (Table 3), these systems are generally built via three principal strategies. One strategy harnesses the intrinsic therapeutic functions of INCs, such as the photothermal effect of GNPs [42], the ferroptosis-inducing capability of FONPs [50], and the calcium-overload-triggered death induced by CCNPs [48], to drive their controlled self-assembly in vitro via host-guest molecular recognition, yielding large aggregates. Enlarging particle size has been shown to promote preferential tumor accumulation through the enhanced permeability and retention (EPR) effect, thereby prolonging intratumoral retention and amplifying their inherent therapeutic activity [75]. Another approach relies on sequential delivery to achieve in situ assembly within tumors. Host nanoparticles functionalized with tumor-targeting ligands are first administered to selectively accumulate at the tumor site. Subsequently, guest nanoparticles are introduced to engage in host-guest recognition, triggering local self-assembly into stable aggregates to enhance retention and potentiate treatment [39]. A third design employs INCs as structural scaffolds for drug carriers, where host-guest complexes are used to load anticancer drugs and achieve controlled release in response to specific signals from TME [55]. A fourth design incorporates INCs into biomembranes or functional polymer matrices via host-guest binding, synergistically improving targeted drug delivery and bioavailability in cancer therapy [41].

**Table 3:** Design, stimuli-response, and performance of host-guest-based INCs.

INCs	Design principles	Stimulus-responsive mechanisms	Performance benefits and limitations	Ref.
GNPs	Host-guest group: CD/ADA	Hydrazone bond: faster hydrolysis at acidic pH	Intratumoral self-assembly; Renal clearance;	[39]
	Targeting group: c(RGDfC)		Low nonspecific accumulation;	
	Stimulus group: hydrazone bond		No clinical translation	
	Host-guest group: β-CD/2-NAA-HA conjugate	Ionization of DOX in acidic pH;	In vitro self-assembly;	[40]
		NIR laser-triggered dissociation	Prolonged tumor accumulation up to 24 h	
	Host-guest group: β-CD/ADA	NIR laser; Synergistic induction of M1 polarization	Intracellular self-assembly;	[41]
	*Escherichia coli* OMVs coated		Inhibited the efflux of immune cells (9.2 h);	
			Elevated toxicity risks	
	Host-guest group:	-	Intracellular self-assembly;	[42]
	CB[7]/Quaternary ammonium-thiol		∼70% intracellular retention	
	CB[7]/ADA			
	Host-guest group: β-CD/ADA	Assembly of amphiphilic nanoparticles upon heating	Thermoresponsive vesicle assembly/disassembly;	[43]
			Lack of applications	
CCNPs	Host-guest group: CB[7]/ADA	Acid-responsive decomposition	In vitro self-assembly; Minimal side effects;	[44]
	Targeting group: VNP		No clinical translation	
	Host-guest group: β-CD/Tocopherol acetate	Acid-responsive decomposition	In vitro self-assembly; ∼95% encapsulation efficiency	[45]
	Host-guest group: CB[6]/hydrophobic model drugs	Acid-responsive decomposition;	In vitro self-assembly; ∼67.2% encapsulation efficiency;	[46]
		Pt-catalyzed H_2_O_2_ decomposition	No in vivo studies	
	Host-guest group: α/β-HPCD/Pd(II) complex	UV-light-triggered release	In vitro self-assembly;	[47]
			Novel catalytic system	
	Host-guest group: CB[7]/FFVLK	SPM overexpression dissociate the CB[7]/Phe	Intracellular self-assembly;	[48]
	Targeting group: folic acid		No obvious side effects	
FONPs	Host-guest group: β-CD/APD	APD exhibits acid sensitivity	Orderly self-assembly;	[49]
	Janus particle		Reusable	
	Host-guest group: CD/ADA	An acidic environment damages the CCM	Intracellular self-assembly;	[50]
	Targeting group: CCM		Ferroptosis	
	Host-guest group: β-CD/ADA	NIR laser;	Bowl-shaped magnetic assembly;	[51]
	Silica coated	Cationic polymers facilitate cellular uptake	Cationic polymers for gene delivery	
	Host-guest group: α-CD/PEG	Fenton catalytic performance;	Supramolecular hydrogel	[52]
		NIR laser		
	Host-guest group: β-CD/Diclofenac	-	Delayed drug release	[53]
SNPs	Host-guest group: β-CD/Azo	Light-responsive Azo isomerization	Interchangeable-engine nanomotors	[54]
	Host-guest group: β-CD/Fc	H_2_O_2_-triggered drug release	Hollow mesoporous silica structure	[55]
	Host-guest group: α-CD/PEG	-	Self-supporting gels	[56]
	Host-guest group: CP5/Py	pH, temperature, EDA, NIR-responsive	High loading capacity;	[57]
	Targeting group: folic acid		Tumor cellular internalization	
	Host-guest group: CD/ADA	DTT reduces the disulfide bonds	∼56.2% drug loading efficiency;	[58]
			∼10.5% drug loading content	

**Abbreviations:** c(RGDfC, Cyclo(Arg-Gly-Asp-d-Phe-Cys); OMV, outer membrane vesicles; VNP, Engineered Salmonella typhimurium VNP20009; CCM, cancer cell membrane.

The design of intracellular self-assembling INCs capitalizes on the synergy between INCs and host-guest chemistry to achieve both therapeutic and diagnostic aims. These interactions fulfill three principal functions: molecular recognition at target sites triggers in situ self-assembly of nanoparticles, increasing their size to improve tumor accumulation via the EPR effect or to amplify treatment outputs [76]. Host-guest binding also allows dynamic conjugation of targeting ligands to nanocarriers, guiding them to diseased tissues and facilitating release in response to local cues such as enzymes or pH changes, thereby improving cellular targeting precision [77]. Additionally, host molecules encapsulate guest-modified drugsto form complexes ,which could be dissociated under distinctive tumor conditions, including acidic pH, elevated enzymes, or high GSH levels, to enable spatially controlled drug release [78].

## 3 Host-guest self-assembly strategies and applications of representative INCs

### 3.1 Gold nanoparticles

GNPs with diameters ranging from several to hundreds of nanometers are widely used as drug carriers due to their biocompatibility, low toxicity, and easy surface functionalization [79]. Ultrasmall GNPs (2-6 nm) have been demonstrated to have superior renal clearance and deeper tumor penetration, which makes them highly attractive for applications in precision oncology [80]. Their compatibility with host-guest systems -could be further improved through straightforward functionalization using thiol-containing ligands or in situ synthesis with macrocyclic hosts [81].

Chen et al. [82] functionalized β-CD-modified GNPs with two ADA conjugates, including ADA-PEG_8_-glycine-arginine-glycine-aspartic-serine, an RGD peptide that targets αvβ_3_ integrin-overexpressing cancer cells, and a hydrazone-linked DOX prodrug (ADA-Hyd-DOX). This strategy yielded a multifunctional nanocomposite with an average particle size of 3.3 nm, where both the targeting peptide and DOX were conjugated through host-guest binding between CD and ADA. The nanocomposite was rapidly internalized by cancer cells to reduce their viability by 30%. By combining passive tumor accumulation with stimuli-responsive drug release, this study illustrates how host-guest chemistry can address key limitations of conventional GNPs-based therapies.

However, the therapeutic efficacy of GNPs in tumor treatment is constrained by insufficient targeted accumulation and short intratumoral retention times [83]. Particles with a size around micrometers could efficiently reduce clearance rates, which often exacerbates nonspecific biodistribution [84]. The aggregation of GNPs could redshift their absorption of LSPR into the NIR region, enabling efficient photothermal conversion [85]. Therefore, intracellular assembly of GNPs into micron-scale aggregates could improve tumor accumulation, extend retention, and enhance therapeutic performance.

Recent efforts have explored stimulus-responsive ligands to direct tumor-specific self-assembly of GNPs for improved PTT. Such systems frequently employ strong host-guest interactions to achieve spatiotemporal control and targeting specificity.

**Fig. 2:**
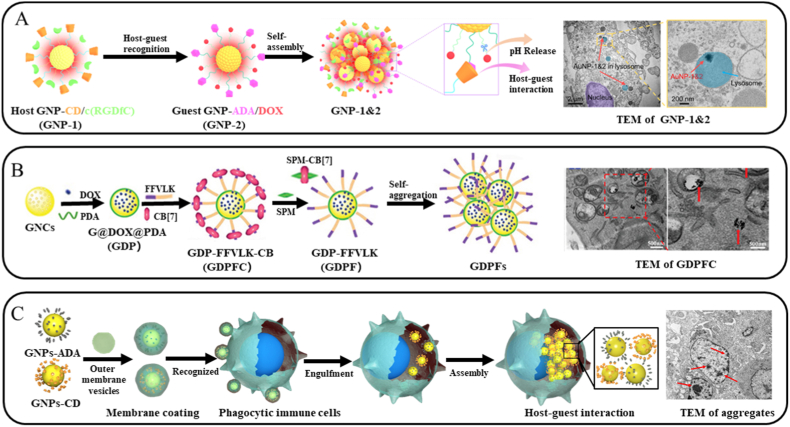
(A) Schematic illustration of GNP-1, GNP-2, and the formation of GNP-1&2 self-assemblies via host-guest recognition and TEM images of the tumor slice after the injection of GNP-1&2. Reproduced with permission [39]. Copyright 2025, American Association for the Advancement of Science. (B) Schematic illustration of the fabrication of GDPFC, the SPM-triggered self-aggregation of GNCs and TEM images of 4T1 cells after 12 h incubation with GDPFC. Reproduced with permission [86]. Copyright 2022, John Wiley & Sons. (C) In vivo construction of immune cell-based nanomedicine carriers and TEM analysis of GNP aggregates in the collected tumor tissues after administration for 24 h. Reproduced with permission [41]. Copyright 2022, American Association for the Advancement of Science.

Yuan et al. [39] introduced a self-aggregation strategy of ultrasmall GNPs at the tumor site via host-guest interactions (Fig. 2A). They firstly functionalized ultrasmall GNPs with c(RGDfC) and CD to form GNP-1, which was injected into mice and showed minimal nonspecific accumulation in liver. Two hours later, the guest modified GNPs (GNP-2) functionalized with pH-responsive hydrazone bond-conjugated DOX and ADA were administered. These two components underwent specific self-assembly within the tumor, achieving a high targeting efficiency (>10.5% ID/g) and markedly increased NIR light absorption, thereby activating a localized photothermal effect. This enhanced photothermal conversion further potentiated chemodynamic therapy, creating a synergistic effect that enabled precise treatment of liver tumors without damaging adjacent healthy tissues.

At present, most methods for the self-assembly of GNPs require surface modification with various compounds, which complicates material preparation and poses challenges for quality control, reproducibility, and practical application. To address this limitation, researchers have exploited differential host-guest affinities to achieve specific self-assembly. For instance, CB[7] exhibits a substantially higher affinity for endogenous SPM than for phenylalanine (Phe), enabling the directed self-assembly of GNPs via peptides terminating in Phe [87].

Using the pentapeptide FFVLK as an example, this segment contains a β-sheet domain and could self-assembles spontaneously into fibrous structures through hydrogen bonding. Sun et al. [88] leveraged this behavior to construct microspheres via host-guest interactions between CB[7] and a drug-conjugated FFVLK peptide, facilitating effective drug encapsulation. Following cellular uptake via endocytosis, the high intracellular concentration of SPM competitively displaces the peptide from CB[7], inducing drug release. Xie et al. [86] extended this strategy by immobilizing FFVLK peptides on GNPs surfaces, where CB[7] binding to the terminal Phe residue could suppresses peptide self-assembly. Upon internalization by cancer cells, intracellular SPM competes for CB[7]to lift the assembly inhibition and promote GNPs aggregation (Fig. 2B). These aggregates subsequently enable NIR light-triggered release of core-loaded drugs, yielding a synergistic therapeutic outcomes.

Gao et al. [41] achieved the self-assembly of GNPs inside cells (Fig. 2C) through encapsulating CD-modified GNPs and ADA-modified GNPs with *Escherichia coli* OMVs. Once introduced into the bloodstream, these OMVs are recognized and phagocytosed by immune cells, the encapsulated GNPs were subsequently released. The liberated GNPs then aggregate within cells via host-guest interactions and accumulate in tumor tissues through inflammatory chemotaxis, enabling synergistic PTT and immunotherapy.

In summary, the incorporation of stimuli-responsive motifs enables tumor-targeted aggregation or intracellular assembly of GNPs based on host-guest interaction into larger structures, which not only allow efficient loading of hydrophobic drugs via the host cavities but also leverage the intrinsic plasmonic properties of GNPs for PTT. Additionally, hybridization with biomolecules such as HA or peptides further facilitates direct self-assembly through specific host-guest recognition. In terms of biosafety, GNPs possess low inherent toxicity, controllable dimensions, and favorable clearance profiles. Certain designs also employ biomimetic components like bacterial membrane vesicles to improve cellular internalization. Future efforts should focus on scalable production, in vivo metabolic pathways, and long-term biosafety evaluation of GNPs.

### 3.2 Calcium carbonate nanoparticles

In clinical oncology, tumor calcification can occur in some patients following radiotherapy [89]. Studies have shown that tumor calcification may be associated with improved survival in colorectal cancer [90]. To induce intratumoral calcification endogenously, researchers have developed a strategy that elevates intracellular Ca^2+^ levels, triggering calcium overload in tumor cells. Excess Ca^2+^ disrupts mitochondrial membrane potential, resulting in mitochondrial dysfunction and ultimately apoptosis [91]. CCNPs are particularly suitable for this approach owing to their high calcium-loading capacity and pH-sensitive behavior. Upon entering the acidic TME, these nanoparticles decompose rapidly to neutralize protons, release abundant Ca^2+^ ions, and produce CO_2_. These products subsequently enter tumor cells and disrupt metabolic pathwaysto induce Ca^2+^ overloading and cell death [16].

Recently, CCNPs-based nanocarriers fabricated through supramolecular interactions have attracted considerable attention. Benefiting from reversible self-assembly, versatile drug-loading capacity, low production cost, excellent biocompatibility, biodegradability, and pH sensitivity, CCNPs-based nanocarriers are widely used as a promising platform for precision calcification therapy. Manabe et al. [45] prepared CD-hybridized porous CCNPs via co-precipitation utilizing the macrocyclic cavities of CD to efficiently encapsulate hydrophobic and ionic compounds, achieving a high drug loading rate (95%).

**Fig. 3:**
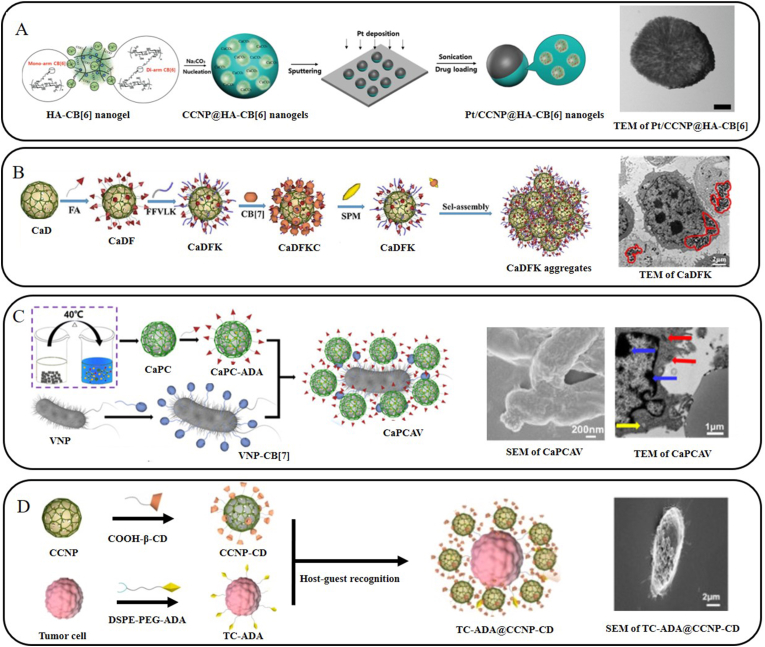
(A) Schematic illustration for the preparation procedures of Janus-type Pt/CCNP@HA-CB[6] nanomotors and HR-TEM image of Janus type Pt/CCNPs @HA-CB[6] nanomotors. Reproduced with permission [46]. Copyright 2019, John Wiley & Sons. (B) Scheme showing the fabrication of CaDFKC nanoparticles and Bio-TEM images of 4T1 cells treated with CaDFKC. Reproduced with permission [48]. Copyright 2023, Elsevier B.V. (C) Scheme of the preparation of CaPCAV, SEM image of CaPCAV, and Bio-TEM images of 4T1 cells after incubation CaPCAV. Reproduced with permission [44]. Copyright 2024, Ivyspring International Publisher. (D) Scheme showing the rapid biomineralization of tumor cells mediated by host-guest interactions and SEM image of TC-ADA@CCNP-CD. Reproduced with permission [92]. Copyright 2025, Elsevier B.V.

Choi et al. [46] also employed a co-precipitation method to synthesize HA-CB[6] -hybridized CCNPs and deposited a thin platinum(Pt) coating on their surface (Fig. 3A). This system exploits the Pt-catalyzed decomposition of hydrogen peroxide to generate propulsive force, enabling autonomous motion of the INCs toward tumors. Under acidic conditions, the CCNPs' core dissolves and releases HA-CB[6] nanogels, which are subsequently internalized by cancer cells via HA receptor-mediated endocytosis. Nevertheless, the drug encapsulation efficiency of this design remains modest (67.2%), and its performance has been evaluated only at the cellular level.

Initial studies primarily focused on leveraging the low toxicity, ease of preparation, and cost-effectiveness of CCNPs in combination with host-guest interactions to construct drug delivery systems. As the development of calcium-overload-induced tumor cell mineralization and calcicoptosis more sophisticated targeting and regulatory strategies have emerged. Xie et al. [48] functionalized CCNPs with folic acid and the self-assembling peptide FFVLK, while blocking the terminal Phe residue of FFVLK with CB[7] to suppress premature self-assembly. Upon cellular internalization, the high intracellular concentration of SPM competitively displaces CB[7], freeing FFVLK to drive intracellular nanoparticle aggregation. This leads to a burst release of Ca^2+^, collapse of mitochondrial membrane potential, and activation of the caspase-3-mediated apoptosis pathway (Fig. 3B).

Beyond small-molecule targeting ligands, anaerobic bacteria such as VNP have been employed as tumor-homing carriers, capitalizing on the hypoxic TME for colonization. Xie et al. [44] utilized host-guest interactions between CB[7] and ADA to coassemble these bacteria with curcumin-loaded CCNPs. In the acidic tumor milieu, nanoparticle dissolution triggers substantial Ca^2+^ release, while curcumin inhibits calcium efflux pumps, synergistically exacerbating mitochondrial dysfunction in cancer cells. Moreover, VNP colonization promotes M1 macrophage polarization and activates adaptive immune responses, thereby amplifying the overall antitumor outcome (Fig. 3C).

To accelerate cellular calcification and circumvent premature degradation of CCNPs during systemic circulation, Xie et al. [92] further devised a rapid cell-surface mineralization strategy. By modifying tumor cells with ADA and CCNPs with CD, host-guest interactions induced the formation of a mineralized coating on the cell surface within 30 min. Implantation of such coated cells into tumor tissue effectively suppressed tumor cell proliferation, invasion and migration (Fig. 3D).

In summary, supramolecular self-assembly based on CCNPs enables modular integration of functional components and efficient drug loading through macrocyclic hosts. Their stimulus-responsive behavior predominantly relies on acidic TME-triggered degradation, resulting in Ca^2+^ release or targeted drug delivery. In terms of delivery efficiency, these systems not only exhibit high drug-loading capacity but can also enhance tumor targeting and retention through surface mineralization and bacterial chemotaxis. CCNPs possess good biocompatibility and well-defined metabolic pathways, the increased biocompatibility can also be achieved through targeting motifs and controlled assembly. Collectively, these features possess CCNPs-based platforms as promising tumor-targeted delivery systems to combine intelligent responsiveness, efficient drug delivery, and favorable safety profiles.

### 3.3 Ferroferric oxide nanoparticles

Magnetic nanoparticles can be manipulated using external magnetic fields. FONPs represent the most extensively studied and applied magnetic nanomaterial, owing to their cubic symmetry, robust stability, low toxicity, and favorable biocompatibility [93]. These characteristics make FONPs particularly suitable for precision drug delivery systems, as they facilitate the targeted transport of therapeutic agents to tumor sites while reducing exposure to healthy tissues [94]. Recent progress in self-assembly techniques has further improved the therapeutic utility of FONPs by exploiting host-guest interactions with macrocyclic compounds such as CDs and CB[n]s [95,96].

**Fig. 4:**
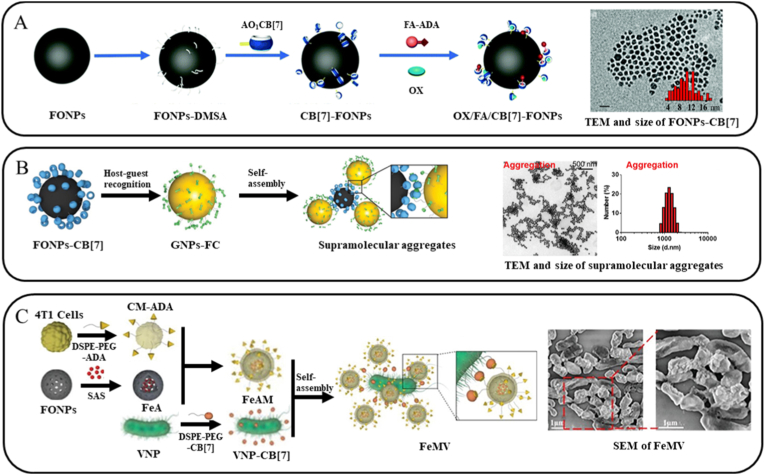
(A) Schematic design of the OX/FA/CB[7]-FONP nanoplatform, and TEM images and size distribution of CB[7]-FONPs. Reproduced with permission [74]. Copyright 2020, Royal Society of Chemistry. (B) Schematic design of Supramolecular tropism-driven targeted delivery and in situ aggregation of GNPs to FONPs, and TEM images and corresponding sizes of supramolecular aggregates of FONP-CB[7] and Fc-GNPs. Reproduced with permission [97]. Copyright 2021, John Wiley & Sons. (C) Scheme showing the preparation of conjugate of CM-ADA coated FONP and CB[7] modified VNP via host-guest interactions, and SEM image of FeMV. Reproduced with permission [98]. Copyright 2023, John Wiley & Sons.

Yue et al. [74] constructed a drug delivery system based on FONPs to achieve efficient drug loading and controlled release via surface functionalization (Fig. 4A). Mono allyl-modified CB[7] (AO_1_-CB[7]) was covalently anchored onto the surface of FONPs through a thiol-ene click reaction. Active targeting was then realized through host-guest interactions between CB[7] and folic acid-modified ADA (FA-ADA), while drug loading was accomplished via the host guest interaction between CB[7] and the antitumor drug oxaliplatin (OX). The anchoring efficiency of FA-ADA was as high as 11.97%, and the loading capacity of OX was 8.02%. Upon cellular uptake, the high intracellular concentration of SPM competitively displaced OX from CB[7], triggering drug release. Within 72 h, the cumulative release of OX reached 70.75%. Owing to the magnetic properties of FONPs, this system also enables MRI, permitting real-time tracking of nanoparticle distribution and accumulation in vivo, thus demonstrating promising theragnostic potential.

FONPs are frequently hybridized with other INCs-to enhance magnetic responsiveness, photothermal properties, and drug delivery functions [99]. Niehues et al. [100] designed a light-responsive GNP-FONP hybrid system by incorporating β-CD with a photo-switchable arylazo pyrazole guest molecule, demonstrating reversible assembly and disassembly under light irradiation.

Cheng et al. [97] leveraged the intrinsic magnetic properties of FONPs to address the inadequate tumor accumulation of GNPs. Their strategy first employed CB[7]-functionalized FONPs as artificial anchors to magnetically deposited at the tumor site. Subsequently, Fc-modified GNPs were guided to these pre-anchored locations via specific host-guest recognition between CB[7] and Fc, enabling on-demand aggregation and precise in-situ PTT (Fig. 4B). The apoptosis rate of B16 cells treated with the supramolecular aggregates increased markedly to 78.7%, compared to only 0.34% in the control group. In vivo antitumor experiments shown that tumor volume was decreased to 63% in the PTT-treated group, demonstrating substantial therapeutic efficacy.

Meanwhile, FONPs have also been combined with porous silica microspheres to construct controlled drug release systems [101]. Indoleamine 2,3-dioxygenase 1 (IDO1) is known to be highly expressed in various cancers, including colorectal, cervical, and ovarian cancer. This intracellular enzyme catalyzes the metabolism of tryptophan to N-formyl kynurenine (F-Kyn), disrupting the host-guest interaction between tryptophan and CB[8] [102]. Hai et al. [103] developed tryptophan (Trp)-labeled porous silica microspheres, and size-matched CB[8]-modified FONPs were further used to block the pores through binding to tryptophan. The system exhibited a high drug loading capacity of 28.6%. Upon cellular uptake, the high intracellular IDO1 expression oxidizes tryptophan, disrupting the host-guest complex and triggering drug release. After 24 h of incubation, approximately 70% of the drug had been released.

FONPs can not only achieve directional aggregation through magnetic control, but also exhibit significant anti-tumor effects. Within the acidic TME, FONPs release Fe^2+^/Fe^3+^ ions that drive a Fenton reaction, generating abundant GSH-dependent ROS and inducing tumor cell ferroptosis with minimal impact on healthy tissues [104].

Capitalizing on this mechanism, Xie et al. [98] constructed a composite system using FONPs (Fig. 4C). They employed VNP as a living delivery vector and encapsulated the ferroptosis inducer sulfasalazine (SAS) into porous FONPs, forming a complex designated FeA. ADA-modified 4T1 cell membrane (ADA-CM) was then prepared and coated onto FeA via ultrasonication, yielding FeAM nanoparticles. Finally, VNPs were functionalized with CB[7] and conjugated with FeAM through host-guest interactions, assembling the FeAMV complex. Following intravenous injection, FeAMV accumulates specifically in tumor tissue owing to the innate tumor tropism of VNPs and the homing capability of the cell membrane coating. Upon cellular internalization, the membrane coating was disassembled during transmembrane transport, exposing FeA and triggering SAS release. SAS inhibits both glutathione peroxidase 4 (GPX4) and the cystine/glutamate antiporter (system Xc^−^), thereby depleting cysteine uptake, suppressing intracellular GSH synthesis, and intensifying ferroptosis. This process not only directly kills tumor cells but also stimulates dendritic cell (DC) maturation and promotes M1 polarization of macrophages within the tumor, synergistically amplifying anti-tumor immunity.

In summary, FONPs with excellent biocompatibility, distinctive magnetic properties and notable photothermal performance enable FONPs to serve as versatile carriers to load hydrophobic drugs via host-guest interactions and achieve spatially precise navigation and site-specific enrichment under external magnetic guidance. Through rational host-guest recognition using macrocyclic molecules, FONPs can be further functionalized with targeting ligands, stimulus-responsive motifs, and therapeutic agents, thereby constituting an integrated and multifunctional delivery platform. Moreover, the inherent magnetism of FONPs allows them to function as MRI contrast agents, enabling real-time theranostic capabilities. FONPs hold strong promise for advancing targeted drug delivery, stimulus-triggered release, and multimodal combination therapy, positioning them as a compelling material platform for the development of next-generation precision oncology strategies.

### 3.4 Silica nanoparticles

SNPs are widely employed in drug delivery owing to their low cost, tunable size, high specific surface area, and abundant surface silanol groups available for modification [[105], [106], [107]]. Supramolecular self-assembly strategies significantly expand the functional versatility of SNPs.

**Fig. 5:**
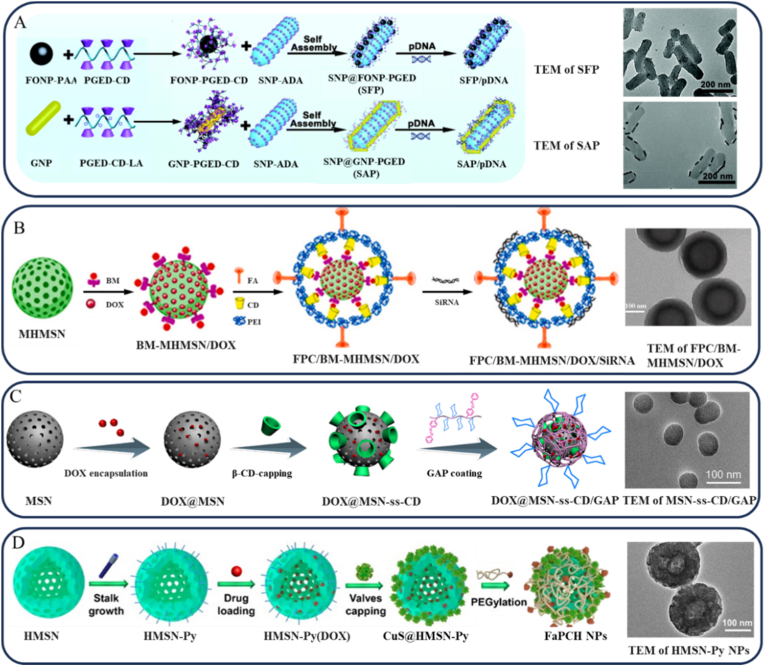
(A) Schematic illustrations of the preparation process of FONP-PGED-CD and GNR-PGED-CD, and the self-assembly of nanohybrids and TEM images of SFP/SPA. Reproduced with permission [109]. Copyright 2018, Royal Society of Chemistry. (B) Schematic illustrations of the preparation process of star-shaped polyethyleneimine-β-CD-coated MHMSN and TEM images of FPC/BM-MHMSN/DOX. Reproduced with permission [110]. Copyright 2023, Elsevier B.V. (C) Preparation of DOX@MSN-ss-CD/GAP for accurate HCC chemotherapy and TEM images of MSN-ss-CD/GAP. Reproduced with permission [111]. Copyright 2023, Elsevier B.V. (D) Schematic diagram for the preparation of the multifunctional supramolecular nanomaterial based on HMSN and macrocycle-capped CuS nano gates, and TEM images of HMSN-Py NPs. Reproduced with permission [57]. Copyright 2019, Elsevier B.V.

Through host-guest molecular recognition, SNPs can self-assemble with complementary functional materials to form hybrid nano systems with controlled structures and reversible dissociation behavior [108]. Serres-Gómez et al. [56] exploited the host-guest interaction between α-CD and PEG-modified SNPs to induce complexation and self-assembly into soft nanogels capable of serving as efficient drug carriers. Duan et al. [109] modified chiral silica nanorods with 3-aminopropyltriethoxysilane (APTES) and ADA carboxylic acid (ADA-COOH), and functionalized FONPs and gold nanorods (GNRs) with CD-conjugated PGED (PGED-CD). Utilizing the specific host-guest interaction between ADA and CD, they constructed a multifunctional nanohybrid integrating PGED with multiple inorganic components. This system was demonstrated to have efficient cellular internalization and enhanced gene delivery efficacy (Fig. 5A).

Additionally, SNPs can achieve controlled drug release via host-guest interactions. Mesoporous silica nanoparticles (MSNs) have attracted particular interest as drug carriers due to their highly ordered porous frameworks [12]. Host-guest interactions facilitate dynamic molecular recognition and binding governed by size and shape complementarity. These non-covalent bonds exhibit moderate binding strength and undergo reversible dissociation in response to external stimuli [112]. Macrocyclic hosts with hydrophobic cavities can encapsulate drugs within MSNs pores and release them upon exposure to specific signals in the TME, enabling precise drug delivery [113].

CDs serve as a key gatekeeper molecule in MSNs systems, owing to their substantial hydrophobic cavity and favorable biocompatibility [114]. Mundžić et al. [115] utilized host-guest interactions between β-CD monoaldehyde and ADA to encapsulate paclitaxel within MSNs channels via acid-responsive hydrazone linkers, enabling drug release in the acidic TME. Similarly, Mousazadeh et al. [110] developed a targeted delivery system based on host-guest interactions between folate-conjugated polyethyleneimine-β-CD (FPC) and benzimidazole (Bzl)-modified magnetic hollow MSNs (BM-MHMSNs) (Fig. 5B). Under acidic conditions, protonation of Bzl reduces its affinity for β-CD, triggering the specific release of DOX.

Light-responsive host-guest systems offer enhanced spatiotemporal control over drug release. Ye et al. [54] reported a supramolecular machine based on Azo and β-CD, where UV light induces Azo's transition from a stable trans-isomer to a metastable cis-isomer, thereby activating drug release from hollow MSNs (HMSNPs). Wu et al. [111] designed a dual-stimuli system by integrating Azo as a photo-responsive gatekeeper with β-CD and galactose for targeting the asialoglycoprotein receptor on hepatocellular carcinoma cells (HCC). This platform enables precise DOX delivery through combined light irradiation and intracellular GSH activation, markedly enhancing therapeutic efficacy against HCC (Fig. 5C).

In recent years, pillararenes have emerged as key molecular gating components in MSN, owing to their electron-rich cavities and highly symmetrical, modifiable structures [116]. Yao et al. [117] employed a water-soluble CP5 to encapsulate drugs within a carrier that dissociates into soluble fragments under acidic conditions, enabling efficient drug release. Innovatively, Xu et al. [118] developed an innovative approach by modifying methyl viologen (MV^2+^) onto MSN surfacesto form a dynamic cap via host-guest interaction with PEGylated CP5. Upon exposure to reducing agents or external voltage, MV^2+^ reduces its neutral form to disrupt the host-guest complex and trigger drug release. Yang et al. [57] constructed a quadruple-stimuli-responsive nanogating system by assembling carboxyl CP5-functionalized CuS nanoparticles (CP5-CuS) with pyridine-modified hollow mesoporous silica nanoparticles (HMSN-Py) through host-guest complexation. In this system, CP5-CuS acts as both a gatekeeper and a photothermal agent. This intelligent design responds to multiple stimuli, pH, temperature, EDA, and near-infrared light, to achieve precise control over drug release (Fig. 5D).

In summary, through supramolecular host-guest strategies, SNPs can undergo precise molecular recognition and assembly with macrocyclic hosts such as CDs and pillararenes, enabling efficient encapsulation of hydrophobic drugs and the construction of smart delivery systems. These systems are responsive to diverse endogenous and exogenous signals in the TME, allowing spatiotemporally controlled drug release. Moreover, hybridization with other functional nanomaterials further enables the integration of multifunctional theranostic platforms, laying a solid technological foundation for the development of next-generation intelligent, efficient, and safe nanomedicines for cancer therapy.

### 3.5 Novel INCs

Advances in materials science and supramolecular chemistry enable a growing number of novel inorganic nanomaterials, such as quantum dots (QDs) and metal-organic frameworks (MOFs), to achieve controllable self-assembly through host-guest interactions, thereby broadening their functional scope and therapeutic potential in oncology.

As nanoscale semiconductor crystals, QDs are primarily composed of group II-VI or III-V elements with typical diameters of 4-12 nm, and exhibit size-tunable photoluminescence and distinct advantages for optical imaging and photodynamic therapy (PDT) [119]. Benefiting from their inherent cytotoxicity against cancer cells, QDs can act as photosensitizers or energy donors in PDT [120]. The supramolecular engineering of QDs could increase the size and enhance drug-loading capacity of QDs, thus improving tumor targeting and retention. For example, Pei et al. [121] prepared a hyper-cross-linked hybrid nanosponge via esterification between carboxylated carbon QDs and β-CD, which achieved a high drug-loading capacity (39.5%) and an encapsulation efficiency (97%) through host-guest inclusion. Yang et al. [122] functionalized graphene QDs with the targeting peptide RGD and PEG-ADA to load drugs onto their polycyclic aromatic structure, which could self-assemble into 60-nm nanotubes via host-guest interaction between β-CD and ADA. In the acidic TME, these nanotubes not only loosened and expanded over 100 nm to significantly prolong the intratumoral retention, but also dissociated into 15 nm particles to promote the internalization by tumor cells.

MOFs are constructed from organic ligands and metal ions or clusters via coordination bonds, possess tunable pore sizes, ordered structures, and high specific surface areas [123]. When macrocyclic hosts such as CDs or CB[n]s are incorporated as organic ligands, the resultant MOFs can conjugate host molecules in a periodic framework to create an ideal environment for molecular recognition and guest encapsulation [124]. For instance, MOFs modified with β-CD (β-CD-MOFs) could be prepared by coordination of potassium ions with β-CD, which exhibited good biocompatibility, straightforward synthesis, and porosity. Hu et al. demonstrated that β-CD-MOFs loaded with ADA-Cy5 or ADA-PEG could preferentially accumulate in tumors through the EPR effect [125]. Although supramolecular MOFs have so far been applied mainly in bioimaging and biosensing, their potential for drug delivery and cancer therapy remains largely untapped due to unverified in vivo safety profiles and metabolic pathways.

In summary, supramolecular modification of QDs and MOFs not only enriches the chemical diversity of self-assembled systems but also opens avenues toward smart delivery platforms with programmable size, tunable fluorescence, and stimuli-responsive behavior. These systems are poised to play an increasingly important role in integrated cancer diagnosis and therapy in the coming years.

## 4 Challenges and prospects

### 4.1 Challenges

Although supramolecularly engineered INCs based on host-guest interactions exhibit promising therapeutic potential in preclinical studies, their translation into clinical cancer therapies still faces several substantial hurdles due to the complex fabrication, biosafety concerns, physiological barriers, and the predictive value of current preclinical models.

#### 4.1.1 Complex fabrication

The fabrication of such intelligent nanosystems typically entails multi-step chemical modifications and precisely controlled self-assembly, rendering the process technically challenging and difficult to scale up with high batch-to-batch consistency [126]. Moreover, unstable physicochemical properties such as particle aggregation, surface oxidation, or degradation can alter key performance parameters, ultimately affecting in vivo behavior and therapeutic outcome [127]. As novel therapeutic products, these systems should also satisfy rigorous quality, safety, and efficacy standards set by regulatory bodies, necessitating comprehensive preclinical characterization and clinical testing, a lengthy and costly endeavor [128].

#### 4.1.2 Biosafety concerns

The distinct physicochemical properties of INCs may lead to unpredictable biological interactions over the long term [129]. Although well biosafety in animal models, their high surface reactivity in the more complex human physiological milieu could provoke nonspecific protein adsorption, immune activation, inflammation, or organ-specific accumulation [130]. Additionally, the safety profiles of external energy sources used in associated therapies, such as laser parameters (power, wavelength, tissue penetration) for photothermal or photodynamic treatment, require careful reevaluation and optimization for human usage [131].

#### 4.1.3 Physiological barriers against effective delivery

From administration to tumor cell engagement, nanocarriers must overcome multiple dynamic biological hurdles [132]. They need to maintain stability in circulation to evade rapid clearance, extravasate through the discontinuous tumor vasculature, and navigate the dense extracellular matrix [133]. After internalization by the cells, most carriers are sequestered in endosomal or lysosomal compartments, -hence the efficient endo-lysosomal escape is critical to prevent payload degradation and ensure therapeutic activity. Failure at any of these steps can significantly compromise treatment efficacy [134].

#### 4.1.4 Limitations of preclinical tumor models

Current research relies heavily on mouse tumor models, which differ fundamentally from human cancers in genetic background, heterogeneity, growth kinetics, metabolism, and immune context [135]. The pronounced drug accumulation and therapeutic responses frequently observed in rodents may not translate to the more heterogeneous and physiologically complex TME in patients. There is an urgent need for more clinical models, such as patient-derived xenografts, organoids, or humanized systems, to better evaluate the translational potential of nanomedicine platforms [136].

### 4.2 Prospects

To overcome the existing challenges and facilitate the translation of host-guest self-assembled INCs from fundamental research to clinical practice, future work should focus on deepening mechanistic understanding, innovating material systems, integrating advanced technologies, and building reliable translational frameworks.

#### 4.2.1 Developing multifunctional synergistic and combined therapy strategies

Currently, liposomes and polymeric nanoparticles are widely used as clinical drug carriers. Although liposomes possess high biocompatibility and versatile drug encapsulation, they suffer from instability, rapid opsonization, limited targeting, and poor batch-to-batch reproducibility, often requiring additional surface engineering [137]. Polymeric carriers have high structural stability and high loading efficiency, but their complex synthesis, potential toxicity, and challenging scale-up with consistent quality limit further application [138]. In contrast, INCs possess superior physicochemical stability, well-defined metabolic pathways, and highly flexible surface-functionalization options. Further biomimetic modifications, such as cell-membrane coating or PEGylation, can enhance biocompatibility, prolong circulation, and enable controlled intracellular assembly, leading to smarter, more efficient, and safer delivery platforms [41,139].

#### 4.2.2 Developing multifunctional synergistic and combined therapy strategies

Future systems can be engineered to respond synchronously to multiple cues in the tumor microenvironment by leveraging the modular nature of host-guest interactions, thereby enhancing targeting specificity and spatiotemporal control. Actively pursuing the deep integration of INCs with advanced therapeutic modalities such as immunotherapy, gene therapy, and metabolic intervention will help establish novel synergistic treatment paradigms capable of overcoming tumor heterogeneity and drug resistance [140].

#### 4.2.3 Advancing functional materials and scalable green manufacturing

The field of biomedical materials is rapidly evolving toward the integration of organic-inorganic hybrids and bio-non-bio interfaces. Further exploration of novel biocompatible materials combined with host-guest chemistry will expand functionalities in areas such as optical therapy, catalytic therapy, and immunomodulation [141,142]. Concurrently, developing simple, safe, cost-effective, and environmentally benign large-scale synthesis and manufacturing processes is essential to transition these systems from the laboratory to the clinic.

#### 4.2.4 Deepening translational research and enabling precision oncology

By employing artificial intelligence and computational modeling, quantitative structure-activity relationships can be established through linking host-guest recognition, stimulus-responsive behavior, in vivo pharmacokinetics, and therapeutic outcomes [143]. This will enable accurate prediction and rational optimization of carrier performance. Strengthening interdisciplinary collaboration across chemistry, materials science, biology, and clinical medicine is crucial to address the engineering challenges in scaling up production under good manufacturing practice standards. Capitalizing on the modularity and adaptability of nano-platforms, integrated diagnostic-therapeutic strategies based on patient-specific biomarkers can be developedto ultimately realize personalized cancer medicine [144].

## 5 Conclusion

In summary, this review highlights the significant potential of intracellular self-assembly of INCs driven by supramolecular host-guest interactions to overcome the limitations of conventional cancer nanomedicine. By combining the unique physicochemical properties of GNPs, CCNPs, FONPs and SNPs, with responsive macrocyclic host systems, intelligent platforms are developed to achieve the precised accumulation, prolonged retention, and controlled drug release in response to specific exogenous or endogenous stimuli.

These supramolecularly engineered INCs not only address critical challenges such as rapid clearance and insufficient intra-tumoral bioavailability but also introduce multifunctionality for synergistic therapy and real-time imaging. The dynamic and reversible nature of host-guest binding facilitates precise spatiotemporal control over nanoparticle assembly and disassembly, thereby improving targeting accuracy and therapeutic efficacy.

Despite such promising advances, several challenges including the scalability and reproducibility of hybrid nano-system fabrication, long-term biocompatibility, and the complexity of the TME still affect the wide clinical application of INCs. Future efforts should focus on developing multi-stimuli-responsive systems, optimizing in vivo performance through detailed mechanistic studies, and combination of therapies with integrate immunotherapy, gene therapy, and diagnostic functions.

We believe that continued innovation in supramolecular chemistry and nanotechnologywill accelerate the clinical translation of these smart INCs, opening new avenues for precision oncology and personalized nanomedicine.

## CRediT authorship contribution statement

**Ying Tang:** Conceptualization, Data curation, Visualization, Writing – original draft, Writing – review & editing. **Yifei Mao:** Conceptualization, Investigation, Writing – original draft, Writing – review & editing. **Luxi Wang:** Writing – review & editing. **Yuan-Fu Ding:** Data curation, Funding acquisition, Project administration, Writing – review & editing. **Peng Li:** Funding acquisition, Project administration, Writing – review & editing. **Beibei Xie:** Conceptualization, Funding acquisition, Project administration, Supervision, Writing – review & editing.

## Declaration of competing interest

The authors declare that they have no known competing financial interests or personal relationships that could have appeared to influence the work reported in this paper.

## Data Availability

No data was used for the research described in the article.
